# A Novel Deep-Learning-Based Bug Severity Classification Technique Using Convolutional Neural Networks and Random Forest with Boosting

**DOI:** 10.3390/s19132964

**Published:** 2019-07-05

**Authors:** Ashima Kukkar, Rajni Mohana, Anand Nayyar, Jeamin Kim, Byeong-Gwon Kang, Naveen Chilamkurti

**Affiliations:** 1 Department of Computer Science Jaypee University of Information Technology, Waknaghat 173 234, India; 2 Graduate School, Duy Tan University, Da Nang 550000, Viet Nam; 3 Department of ICT Convergence Rehabilitation Engineering, Soonchunhyang University, Asan 31538, Korea; 4 Department of Information and Communication Engineering, Soonchunhyang University, Asan 31538, Korea; 5 Department of Computer Science and IT, La Trobe University, Melbourne 3086, Australia

**Keywords:** software reliability, severity classification, deep learning, natural language processing, n-gram, convolutional neural network, random forest

## Abstract

The accurate severity classification of a bug report is an important aspect of bug fixing. The bug reports are submitted into the bug tracking system with high speed, and owing to this, bug repository size has been increasing at an enormous rate. This increased bug repository size introduces biases in the bug triage process. Therefore, it is necessary to classify the severity of a bug report to balance the bug triaging process. Previously, many machine learning models were proposed for automation of bug severity classification. The accuracy of these models is not up to the mark because they do not extract the important feature patterns for learning the classifier. This paper proposes a novel deep learning model for multiclass severity classification called Bug Severity classification to address these challenges by using a Convolutional Neural Network and Random forest with Boosting (BCR). This model directly learns the latent and highly representative features. Initially, the natural language techniques preprocess the bug report text, and then n-gram is used to extract the features. Further, the Convolutional Neural Network extracts the important feature patterns of respective severity classes. Lastly, the random forest with boosting classifies the multiple bug severity classes. The average accuracy of the proposed model is 96.34% on multiclass severity of five open source projects. The average F-measures of the proposed BCR and the existing approach were 96.43% and 84.24%, respectively, on binary class severity classification. The results prove that the proposed BCR approach enhances the performance of bug severity classification over the state-of-the-art techniques.

## 1. Introduction

**Figure 1:**
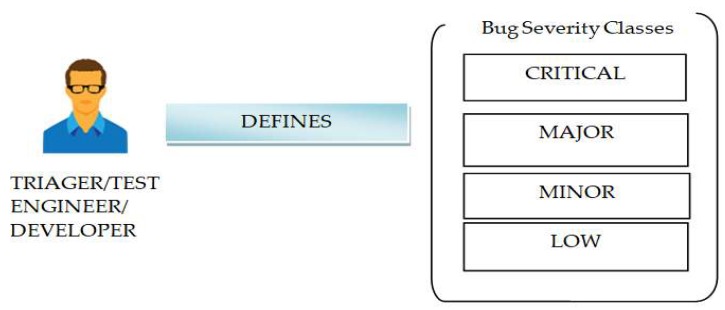
The basic classes of bug severity.

With the constant expansion of modern software, its reliability has become questionable, as these programs are prone to many problems and even failure. Software bugs are one of the enduring issues in the software development process. These are created in software during the development process by a programmer’s mistake, such as memory flow issues, run-time issues, and deviations from the pre-coordinated running directions [1]. These software bugs can trigger significant security issues, particularly in the area where the fault tolerance rate is low. For example, the Heart Bleed Bug attacked the encryption systems of the software industry in 2015. Therefore, the bug’s presence proved quite costly. As the budget of the software industry is limited, it is helpful first to track a bug and evaluate its severity at the right time. In standard practice, the end user experiences a bug while dealing with the system and reports the bug in the bug tracking system (BTS) by entering the severity level, description, product, platform, and component fields. The developer (triager) uses this information to resolve the costly bug, which is a time-consuming process. The triager has to choose and identify the issue from a large number of communicated software bugs [2,3,4]. Different bugs have different impacts upon the quality, based on the functionality of software. The triager (test engineer or developer) assigns the different severity classes to the bug report, as shown in Figure 1. Not all bugs relateto critical issues, as some are frivolous and appeal for an improvement [5,6]. Let us take an example of anemail service provider (ESP). If the system crashes or tosses the error message in spite of signing in with the correct password and user name, such a bug is classified as a critical bug, as it makes the entire application unusable. Further, if there is a major functionality issue, such as when the carbon copy (CC) section of the ESP does not allow adding more than one recipient, then it is also classified as a major bug because it makes the application dysfunctional. The “Terms and Conditions” option in the ESP has multiple links, and if one link is not working properly among the various links, then it is labeled as a minor bug. It does not affect the usability of the application and if there is a spelling mistake in the “license page” of the ESP, this type of issue is classified as a low priority bug.

In the case of numerous bug reports, the manual severity classification is a very dull, laborious, and time-consuming task. Manual bug severity classification is performed by utilizing the bug report content (summary and description). Therefore, bug severity classification techniques that automatically classify the bugs according to comments have generally been utilized by developers to dispense their limited resources.

On the other hand, the bug severity also acts as a dispute between user and developer which requires prompt consideration and resolution. It is a fundamental characteristic that gives general advantages to the industry, which requires earnestness in its resolution with respect to resources. It proves that 80% of bug reports goes through severity reassignment, even after the bug report has been sent to a developer [4]. Additionally, it demonstrates that bug reports with severity reassignment have fundamentally longer resolution times than those with no reassigned severity. These bug severity fields are utilized by the triager to assign the appropriate development groups. The inaccurate severity assignment essentially postpones the processing time of the bug report, thus influencing the efficiency of developers’ work [7]. Numerous bug severity classification techniques have been proposed [8,9,10,11]. Classification models extract the bug features from bug reports, which act as inputs and use various machine learning algorithms for training purposes. After that, the model is used to classify the severity of a new bug report. The researchers in the field of severity classification focus on two main points: (1) extraction of features related to bugs via manually designed combination; and (2) to improve the accuracy rate by developing an effective classifier using various machine learning algorithms.

### 1.1. Motivation Example 

**Figure 2:**
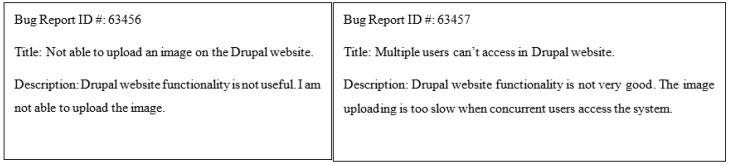
Example of bug reports.

Automatic bug severity classification can be formulated as a classification problem using the bug report content. However, the information (content) in the bug report has semantic and syntax structure and comes with feature representation and non-linearity issues, which previous feature extraction techniques have failed to resolve. The classifier learns the feature representation of the corresponding class label during the training phase. Previous studies have used the “bag of words” or term frequency for feature representation, which misclassified the model [12,13,14]. The reasons for misclassification are: (i) the model considers the sentences as a stack of words that has an unordered sequence; (ii) it does not consider the semantic similarity between the words and comprehends the meaning of slightly variant sentences as the same. For example, in Figure 2 the sentence in Bug ID 63456 reads “Drupal website functionality is not good”, and in Bug ID 63457 it reads “Drupal website functionality is not very good”. Both sentences have approximately the same “bag of words” and are classified in the same class, but Bug ID 63457 is not as critical as ID 63456.The previous n-gram approach also considers the same word order, which increases the dimensionality and sparsity of the data, therefore the classifier model becomes more complicated by enhancing the computation. Further, these feature extraction techniques do not capture the latent features [15,16] because they work on the linear equation and have a fixed length of polynomial terms. Moreover, the traditional bug severity classification model operates under shallow architecture due to the constrained generalization capability and overfitting issues in the multiclass classification process. Hence, the severity classification results of the previous method are not up to the mark.

This motivates us to utilize the deep learning model. This model enhances the generality by using a high number of kernels on the filter. In spite of this, the deep learning technique has proven to be a powerful technique for automatic feature extraction and has multiple layers within the deep architecture. It can capture highly complicated and important non-linear (latent) features. It also classifies more complex data rather than shallow techniques by utilizing the intermediate multiple hidden layers [17]. To utilize its powerful ability of extraction and learning, the advanced method Convolutional Neural Network leverages the learning of semantic features from the token vectors extracted from the bug report text. Recently, a Convolutional Neural Network (CNN)-based deep learning technique changed the concept of feature representation and exhibited a promising breakthrough in software bug prediction in source code [18]. The deep learning researchers reported that the CNN is more advanced than the Deep Belief Network (DBN)or the Recurrent Neural Network (RNN) [19] in effectively capturing the local patter in the application of Speech Reorganization (SR) [20,21], Image Classification (IC) [22,23,24], and Text Classification [25]. Thus, it is more capable of finding local patterns and conducting severity classification.

### 1.2. Research Contribution 

Learning the latent features and semantic representation of features from the text of the bug report, preserving the word order, and removing the sparsity and overfitting of data are challenging research problems in bug severity classification. Thus, this paper proposes a deep learning technique called Bug Severity classification using a Convolutional Neural Network and Random forest with Boosting (BCR), which can learn the feature representation directly without the need for manual feature engineering. It also captures semantic and non-linear feature mapping of the bug report text. Firstly, the text of a bug report is preprocessed followed by application of the n-gram technique to extract the n-gram, and then the CNN is applied to extract the latent and high representative features. Eventually, the random forest with boosting is applied to classify the multiple bug severity classes. The results prove that the proposed BCR approach enhances the performance of bug severity classification over the state-of-the-art techniques [26].

This research paper seeks to address the following research questions:

- Whether it is feasible to implement automated multi-bug severity classification model using deep learning technique and what the best configuration of Bug Severity classification is via Convolutional Neural Network and Random forest (BCR);
- Whether the proposed algorithm outperforms the Traditional machine learning algorithms in bug severity classification.

This paper utilizes the following key contributions:

- To the best of our knowledge, we have taken the first step to build the multiclass bug severity classification model via deep learning and random forest for open source projects;
- The n-gram technique is employed to generate the features from the text, which captures word order and the characteristics of different severity classes in five open source projects;
- A CNN-based feature representation framework is proposed to automatically generate the semantic and non-linear mapping of features, which preserves the semantic information of the bug report text;
- The CNN and random forest with boosting acclimates for severity classification instead of the traditional machine learning algorithms. The evaluation results show that the proposed algorithm outperforms the other algorithms in binary and multiple bug severity classification.

The present study is categorized into six parts. Section 2 presents related work about bug severity classification. Section 3 illustrates the methodology of the proposed BCR model. Section 4 demonstrates the experiment’s results and the answer to research questions. The possible validity threats of the proposed model are presented in Section 5, and Section 6 discusses the conclusion.

## 2. Related Work

Bug severity classification is an active research field in software engineering (SE). The spotlight of previous bug severity classification algorithms has been mainly on the manual arrangement of new discriminative techniques and the combination of features from the labeled severity data of bug reports. These features are fed into the machine learning classifier to detect severity. Commonly, the severity is classified into binary (BC) and multiple classes (MC).

### 2.1. Binary Classification 

Zhou et al. [26] proposed a hybrid approach to automate the classification process. They combined data and text mining techniques. Initially, the bug report summary was extracted by using the text mining techniques, followed by their division into three levels. Then, in the second stage, other structures and features were extracted and supplied to the machine learner. Various data grafting techniques were utilized to combine these two stages. The performance showed the best results by increasing the f-measure from 79.8% to 85.9% for OpenFOAM, 84.6% to 93.7% for JBoss, 78.3% to 81.7% for Mozilla, 76.7% to 80.2% for Eclipse, and 72.8% to 79.5% for Firefox. Jin et al. [27] used the same dataset that was used in a previous study [28] and ended up with better results. A classifier model using naive bayes (NB) in addition to the text and meta-fields of the normal bug severity report was designed. In other studies, the use of normal bugs and added fields were not taken into consideration. The F-measures of Eclipse and Mozilla datasets were calculated as 80% and 83%, respectively. Pandey et al. [14] applied different machine learning algorithms like NB, Linear Discriminant Analysis (LDA), Support Vector Machine (SVM), Random Forest (RF), and k-nearest neighbors (K-NN) to classify the bugs into severe and non-severe categories. The authors reported that the performance of the classifier depends on the dataset and achieved accuracy in the range of 75% to 83%. Goseva et al. [29] implemented supervised and unsupervised approaches to classify security and non-security bugs of a National Aeronautics and Space Administration (NASA) dataset. The term frequency—inverse document frequency (TF-IDF), Term Frequency (TF), and Bag of word frequency feature vector methods for both approaches were used. Further, multiple classifiers, such as Naive Bayes Multinominal (NBM), SVM, K-NN, NB, and Bayesian Network BN for supervised approach and anomaly detection method for the unsupervised approach were utilized. The results revealed that the supervised approach performed better than the unsupervised approach. In our previous study [30], we proposed a hybrid approach by using TF-IDF and bi-gram feature weighting methods to increase the performance of the K-NN classifier. The study explored four additional fields with the textual field in training and testing data. The performance was measured by using the F-score. It was observed that the performance is dependent on the dataset.

### 2.2. Multiclass Classification

Jindal et al. [31] used the text mining approach to extract the relevant features of various Payload Integration Test Set (PITS) datasets of NASA projects. The prediction was made by using the Multi-nominal Multivariate Logical Regression (MMLR) statistical method, Multilayer Perceptron (MLP), and the Decision Tree (DT) machine learning algorithm. Among all the methods, DT gave the best result. The three machine learning techniques (NB, SVM, and K-NN) and the seven projects of Eclipse were used [32]. The bagging and vote technique to extract the summary of the bug reports was used. The results showed that K-NN performance was better than the SVM performance. The performance of NB was deficient, i.e., below 34.25% F-value. K-NN provided the best training candidates who helped in cross-project bug severity prediction by combining more than one training sample. In addition, Zhang et al. [33] also modified the REP and K-NN methods to find the bug report, similar to the historical bug report. Further authors extracted the features to classify the bug reports into Blocker, Trivial, Critical, Minor, and Major classes of Eclipse, GCC bugzilla, and Mozilla datasets. It was observed that the similarity measure improved the severity prediction and fixer recommendation process. Sharmin et al. [34] drafted a bug feature selection (BFS) technique by using Pareto optimality to classify the bugs into Blocker, Trivial, Critical, Minor, and Major classes by searing informative features. The performance was evaluated on three open source projects, i.e., Eclipse, GCC, and Mozilla, and results showed that the BFS technique performed better than existing techniques [33]. Kumara et al. [35] implemented a severity classification technique by using NB, KNN, J48, RF, Relative Neighbor Graph (RNG), Condensed Nearest Neighbor (CNN), and Multinomial Logistic Regression (MLR) techniques with entropy. The best results showed increases of accuracy from 61.82% to 75.71% for Eclipse, 86.52% to 98.90% for PITS, and 66.11% to 82.70% for Mozilla.

The literature review posits that various feature extraction techniques, such as TF-IDF, n-gram, In-fogain, HAD, BOW, and KL-divergence, were utilized. The selection of the feature extraction technique depends on engineering design, and it is a challenging task because the best feature representation for collecting data depends on the feature model. In this paper, this challenge is addressed, and a CNN algorithm has been designed. It precisely learns the best feature representation from the bug report data in a supervised fashion. The NLP applications and n-gram technique are used to capture the word order, which helps improve the performance.

## 3. Research Methodology

**Figure 3:**
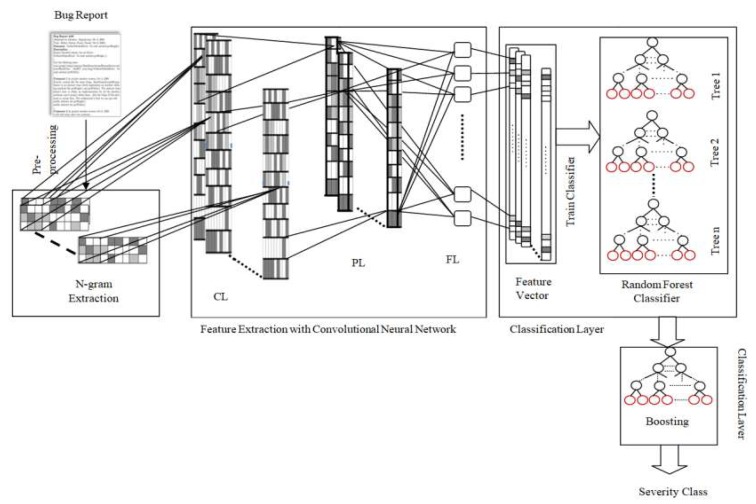
Bug Severity classification using a Convolutional Neural Network and Random forest with Boosting (BCR) model: an n-gram as the n-gram feature extractor, CNN as the feature extractor, and random forest with boosting as the classifier.

In this section, the proposed BCR approach is elaborated. This framework automatically generates the features from the content of bug reports for accurate bug severity classification. The overall framework of BCR is illustrated in Figure 3.

### 3.1. Overall Framework 

**Figure 4:**
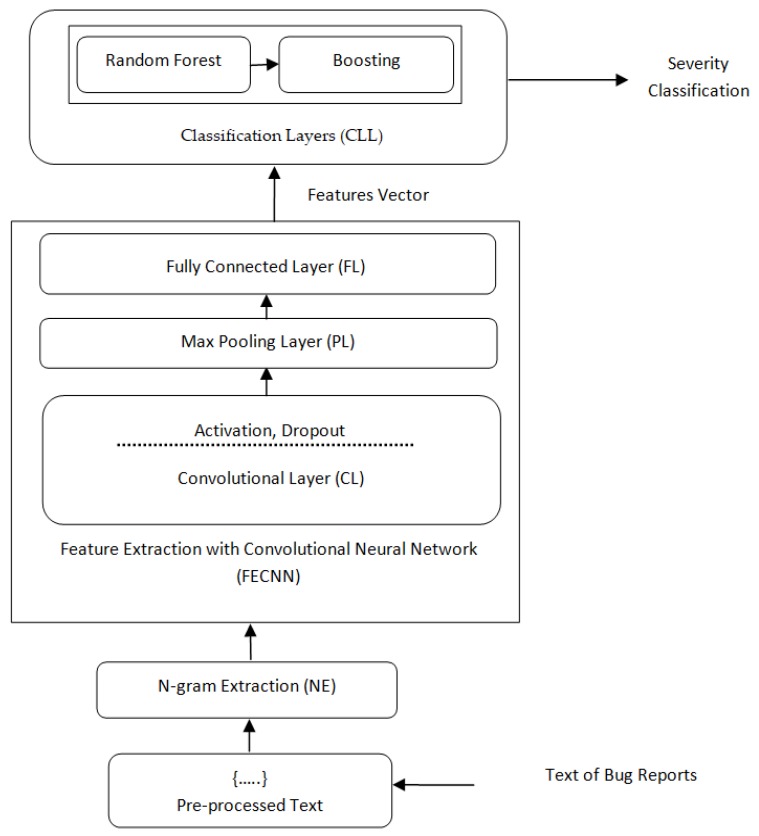
The steps of the BCR model for bug severity classification.

The problem of automatic bug severity classification is defined as the supervised classification algorithm. Figure 4 highlights the essential steps of the proposed BCR model. The steps are explained as follows:

- The bug reports with a title, description, and summary are considered for the input, and the code snippets, URLs, hexcode, and stacktraces of the bug report are removed;
- As seen in Figure 4, the initial step is preprocessing; in this, each word of the bug report content is converted into more manageable representation (Section 3.2). The preprocessed content goes through the next step, which is n-gram Extraction (NE). In the NE step, a set of n-gram features (words) are extracted, which enlarges the word integer into the Feature and Label matrix, which illustrates them in a meaningful way (Section 3.3). The output of NE goes into the Feature Extraction with Convolutional Neural Network (FECNN). It learns semantically meaningful representations of the sentences. It is consists of seven hidden layers. Each hidden layer contains a convolutional layer, max pooling layer, activation function, dropout layer, and fully connected layer. Firstly, the features are extracted by the CNN in the convolutional layer (CL), which extracts and transforms the features by max pooling (PL). Further, the fully connected layer (FL) combines all the features to develop feature space (Section 3.4). Dropout layers and activation functions are utilized before and after the convolutional layer. In the first three layers, the sigmoid activation function is used, and in the last tanh, the activation function is used. It gives the output as a feature vector, and these are fed into the classification layer (CLL), which is utilized to classify the severity of each bug report (Section 3.5). Section 3.5 is divided into three further sections. The initial Section 3.5.1 explains the full process of feature extraction via CNN and random forest with boosting classifier (CNRFB). Section 3.5.2 and Section 3.5.3 illustrate the training process of CNRFB and the process of random forest with boosting classifier (RFB).
- The bug report dataset is divided into training and testing with 10-fold cross-validation to avoid the training biases. The supervised classifier is trained with the features that are extracted by the FECNN module in the training part. In the testing part, the extracted features are used by the trained classifier for severity classification.

### 3.2. Preprocessing 

The preprocessing step aims to remove the unnecessary words from the content of the bug report. The words that are not useful for analyzing the data are removed because their presence can degrade the learning performance. The feature set space is reduced by removing unnecessary words, making it effortless for learning techniques, so as to execute data analysis [36]. This includes three necessary steps: the first is tokenization, the second is stop-word removal, and the third is stemming. Initially, the stream of text is broken into words, numbers, punctuation, and so forth, called tokens. Further, all the punctuation is replaced with blank spaces, non-printable escape characters are removed, and the words are converted into lowercase. After that, the stop words, such as verbs, nouns, articles, pronouns, adverbs, prepositions, and so forth, which are provided in the Natural Language Toolkit, are expelled. Finally, the most essential step is performed, called stemming. In this, the common stem of the words is replaced and saved as a selected feature. For example, the word “move”, “moves”, “moved”, and “moving” are replaced with “move”. The words which are gained after preprocessing are called features, as shown in Equations (1) and (2).

(1) $$BR=\left\{S_{1},S_{2}\ldots \ldots .S_{j}\right\}$$

(2) $$S_{j}=\left\{f_{1},f_{2}\ldots .f_{k}\right\}$$

### 3.3. N-Gram Extraction 

During feature extraction, the content of the bug report is represented as a vector of feature (word) counts and helps in expanding the network by transforming bug report content into several sets of n-gram data. Hence, the features are expressed in a meaningful way. The n-gram technique is implemented to capture the semantic relationship and calculate the frequency of the feature order [37]. The n-gram model is truncated as *k*−1 and characterizes the sequence of *i* features into unigram, bigram, and trigram.

(3) $$p\left(f_{i}|f_{1},\ldots \ldots \ldots .f_{i-1}\right)=p\left(f_{i}|f_{i-k+1},\ldots \ldots ,f_{i-1}\right)$$

In Equation (3),$f_{i}$ is the feature (word) and p() is the probability.

We assume that in the unigram, the next features do not depend on each other and features in the feature string do not have any mutual information. Therefore, the conditional probability of the unigram is as follows:

(4) $$p\left(f_{1}^{k}|\omega_{1}\right)=\prod p\left(f_{i}|\omega_{1}\right)$$

In Equation (4), $\omega_{1}$ is the assumption in which features are independent.

In the bigram, two contiguous features (another specific feature follows one feature) have language information. The conditional probability of the bigram is given by:

(5) $$p\left(f_{1}^{k}|\omega_{2}\right)=\prod p\left(f_{i+1}|f_{i},\omega_{2}\right)$$

where $\omega_{2}$ is the assumption in which two adjacent features are dependent.

The trigram assumption is similar to the bigram; three contiguous features are considered for conditional probability and expressed as:

(6) $$p\left(f_{1}^{k}|\omega_{3}\right)=\prod p\left(f_{i+1}|f_{i},f_{i-1},\omega_{3}\right)$$

In Equation (6), $\omega_{3}$ is the assumption in which one feature is relative to its two predecessors’ features.

Different n-gram models should be combined to exploit its full capacity. The different n-gram models are employed to analyze an individual sentence, and subsequently, analyses of the results are combined. Therefore, the relationship between each n-gram feature is addressed as a word-level analysis as follows:

(7) $$p\left(f_{1}^{k},f_{k+1}\right)=\sum p\left(f_{1}^{k}\right)p\left(f_{k+1}|f_{k}\ldots f_{k-m},\omega_{i}\right)p\left(\omega_{i}\right)$$

where $f_{1}^{k}$ represents the mean of the feature string that contains n features, $f_{k+1}$, (*K*+1)th is the feature, $\omega_{i}$ presents the assumption i, $p\left(f_{k+1}|f_{k}\ldots f_{k-m}\right)$ demonstrates the probability of the feature string, and $p\left(\omega_{i}\right)$ is the probability of assumption i. The extracted features are fed into the CNN model.

### 3.4. Feature Extraction with Convolutional Neural Network (FECNN)

The main operation of the convolutional layer is scanning of the sample data space with various kernels. These kernels demonstrate the various concerned features. CNN can reduce the data size by extracting the highly complicated and important non-linear features. Therefore, the convolutional layer is designed to compute 2D convolutional features. It is divided into a further five layers, which are the Input, Convolutional, Activation, Dropout, Max pooling, and Fully connected layers, as shown in Figure 4.

The Input layer contains the pre-processed data and the n-gram representation of the sequence features. These features are fed into the convolutional layer to generate new feature maps. Let $t_{j}\in {\mathbb{R}}^{i}$ be the *i*-th dimensional feature corresponding to *j*-th feature in the sentence. The representation of the full sentence with length n is:

(8) $$t_{1:n}={\;t}_{1}⊕t_{2}\ldots \ldots ⊕t_{n}$$

where ⊕ represents the concatenation operator; further, it executes the filter operation $\in {\mathbb{R}}^{gi}$, where w is the weight matrix (filter) that is applied to the window of g features to produce the new feature. Take the window of features $t_{j:j+g-1}$ to generate the new feature $f_{i}$:

(9) $$f_{i}=l\left(w\cdot t_{j:j+g-1}+b\right)$$

where b serves as the bias term and b∈ℝ, l is the non-linear function, called the sigmoid. This operation is executed for all possible windows of features in the input sequence $\left\{t_{1:g},t_{2:g+1},\ldots .,t_{n:-g+1:n}\right\}$ and builds a new feature map:

(10) $$f=\left[f_{1},f_{2},\ldots .,f_{n-h+1}\right]$$

The convolutional layer takes the input size [1000 × 200]. The FECNN model uses neurons with the following features: stride S = 1; depth K = 128; filter size F = 20; and no padding P=0. The output size of a CL is equal to ((input width–filter size)/stride + 1 = 90). Every 90 × 200 neurons are associated to a size (1 × 32) of the input region. Furthermore, all 200 neurons in each depth column link to the same (1 × 32) of the input region, with different weights.

The convolutional layer is composed of convolutional kernels that produce feature maps; it is followed by the max pooling kernels. The operation of the max pooling layer concentrates the feature map by reducing the size of the feature map. It is achieved by computing the max value. Hence, the valid information is passed from the previous to the next layer by activating the consecutive layers over complex features. Therefore, overfitting is reduced. The max pooling procedure executes reduction of the input dimensions to 1 × 32 × 200–1 × 1 × 200 and reduces each (1 × 32) feature map to a single number by exerting the average of all 1 × 32values.

To reduce the co-adaption issue in the FCNN model, the dropout layer is introduced, so as to disconnect neuron connections between the connected layers at a specific dropout rate. Therefore, training samples are generalized efficiently by the network. In this research work, the dropout rate is 0.2.

Additionally, the activation function is introduced to decide which neurons must be activated and if the receiving information is relevant or should be ignored. Thus, all the layers are non-linear in the neural network; an activation function computes each neuron value. In this research work, the sigmoid activation function is used in the first 4 layers and the tanh activation function is utilized in the last 3 layers.

The fully connected layer neurons have the entire association with all activations in the previous layer. This is connected with the FECNN module, where feature vectors are combined to develop the feature space for the classification layer.

### 3.5. FECNN with Random Forest with Boosting Classifier (CNRFB)

#### 3.5.1. CNRFB Model 

In this paper, the CNN layers automatically extract the high representative and latent feature. However, in traditional CNN, the fully connected layer acts as a classifier, which computes activations and accumulation as the final decision. However, in this work, the Random Forest (RF) is used as a classifier, since it is more reliable, highly accurate, and more robust than the fully connected layer due to the number of decision trees and bagging strategies participating in the process. It also overcomes the overfitting problem because it utilizes the all prediction average, which cancels out the biases [38]. The proposed BCR model effectively associates with the feature extraction and the classification abilities of CNN and RF, which means the CNN layers acts as feature extractor and RF acts as a classifier in the BCR model. The fully connected layer provides the output in the form of the feature vector, as shown in Figure 3. The dimensions of these feature vectors are equal to the number of neurons in a fully connected layer. For a feature, $\left(\vec{F},L\right)$,$\vec{F}$ contains input features and L defines the severity class. The output layer uses the feature vector $t_{x}$ for computing the severity class $L_{o}$. Based on L (predictive class) and $L_{o}$ (actual class), an error value can be calculated, as shown in Equation (11).

(11) $$E=e(||L-L_{o}||)$$

where e(x) represents the loss function. At that point, the entire network is back propagated through E, and the gradient descent method is used to adjust all the weights. Once the convolutional layers have been trained, the whole sample of the training dataset $\left\{\left(\vec{F_{1}},L^{1}\right),\left(\vec{F_{2}}.L^{2}\right),\ldots ,\left(\vec{F_{k}},L^{n}\right)\right\}$ can be supplied to the network and its analogous output from a fully connected layer $t_{x}^{1},t_{x}^{2},t_{x}^{3},\ldots ,t_{x}^{n}$ and its class $L^{1},L^{2},\ldots ,L^{n}$ can be arranged as the new training dataset $\left\{\left(t_{x}^{1},L^{1}\right),\left(t_{x}^{2},L^{2}\right),\ldots .,\left(t_{x}^{k},L^{n}\right)\right\}$, with which the RF classifier can be effectively trained. In this way, the BCR model is introduced for bug severity classification, as illustrated in Figure 3. The unigram, bigram, and trigram features are arranged together in the FECNN sample, which fuses semantic relationships of features and word order information, and then the sample is used by the FECNN model to extract high representative features for the RF classifier.

#### 3.5.2. Training Procedure of CNRFB

As described in the previous section, considering the feature, instead of training the RF classifier with raw (initial) data of bug reports $\left(\vec{F},L\right)$, a feature vector $\left(t_{x},L^{1}\right)$ is extracted by Convolutional layers to train the RF Classifier. To achieve such feature, vectors can be utilized as training data for the RF classifier; there are two main tasks for training, the first one is to feed the features in the FECNN model that are produced by the n-gram technique, and the second is to train the RF classifier with the features that are extracted by FECNN. The cross-entropy is utilized as the loss function in the FECNN model, which is defined by:

(12) $$E=-\frac{1}{n}\overset{n}{\underset{i=1}{\sum }}\left[L^{i}\log L_{o}^{i}+\left(1-L^{i}\right)\log\left(1-L_{o}^{i}\right)\right]$$

where $L_{o}^{i}$ is the output result of *i*-th neuron in the output layer;$L^{i}$ represents the corresponding target result. After the contribution of the weight’s component has been back propagated on the final *E*, every weight value can be adjusted https://www.powerthesaurus.org/in_accordance_with/synonymsby the *E* value in the network. The contribution of the weight’s component is stated by the partial derivative of *E*, which has the weight’s value *v* and is expressed as $\frac{\partial E}{\partial v}$. This value can be determined through the chain rule by the following equation:

(13) $$\frac{\partial E}{\partial V}=\frac{\partial E}{\partial u}\cdot \frac{\partial u}{\partial v}$$

where the output value *u* of the corresponding neuron is calculated by using the sigmoid activation function and by using the chain rule,

(14) $$\frac{\;\partial E}{\partial v}=x\left(L-L_{o}\right)$$

Moreover, the value of *v* is updated by using the gradient descent rule, as illustrated in the following equation:

(15) v=v+Δv

where,

(16) $$\Delta v=\lambda \frac{\partial E}{\partial v}$$

where λ is the descent learning rate. The convolutional layers can be well-trained with multiple training rounds according to gradient descent and backpropagation method for a specific task.

After the training of convoluting layers, all training data sets $\left\{\left(\vec{F_{1}},L^{1}\right),\left(\vec{F_{2}}.L^{2}\right),\ldots ,\left(\vec{F_{k}},L^{n}\right)\right\}$, where 5≤n≤8,are supplied to the network. The output of every neuron is extracted for each sample $\left(\vec{F},L\right)$ and the array $t_{x}$ is composed. This array illustrates the extracted features that correspond to the raw sample data. The new training dataset $\left(t_{x},L\right)$ is generated with the class L. The new dataset $\left\{\left(t_{x}^{1},L^{1}\right),\left(t_{x}^{2},L^{2}\right),\ldots .,\left(t_{x}^{k},L^{n}\right)\right\}$ is generated after all the training samples have been supplied to the network. With this training dataset, the random forest is trained with the boosting strategy; thus, a BCR model is efficiently trained, where 2-D data are composed of n-gram features. In the proposed BCR model, the FECNN model is employed to extract the high representative features from the output of the feature weighting, and these features are utilized by the RF classifier for the bug severity classification. For the bug severity classification of a new bug report, it is fed into the FECNN after the preprocessing and n-gram extraction, and then the fully connected layer is produced with the highly represented features that are fed into the well-trained RF classifier. The decision of the RF classifier after the boosting is considered as the output of the BCR model. The training process of CNRFB is explained in Algorithm 1.

**Algorithm 1:** The Training Process of CNR Model **Input**: Training Bug Report Dataset BRD= $\left\{\left(F_{1},L_{1}\right),\left(F_{2},L_{2}\right),\ldots ,\left(F_{n},L_{n}\right)\right\}$ ; Maximum Iteration:MI; Threshold:δ; **Batch Size**: BS; Iteration Time: IT **Output**: CNRFB model *cnrfb_model*
Initialize the FECNN model *fecnn_model*While (IT < MI && error> δ):// get the batch of training samples randomlyThe new BRD’ = *fecnn_model*. get batch data (b_size,BRD)// forward the signle and calculate the errorerror = *fecnn_model* -> forward (BRD’)// back propagation the error and modify the weights*fecnn_model* -> batch gradient decent (error)// get the features outputted by fully connected layer$BRD^{feature}$ = *fecnn_model* -> forward get features (BRD)Initialize the random forest with boosting *rf_ classifier*// train the *rf_ classifier* with the extracted features*rf_ classifier* -> Train ($BRD^{feature})$*cnrfb_model* = construct _*cnrfb*(*fecnn_model*, *rf_ classifier*)// construct_*cnr* (*fecnn*, *rf*):Classify (sample S):$S^{feature}$= *fecnn*. forward get features (S)return severity class = *rf* ->classify($S^{feature}$)


#### 3.5.3. Random Forest with Boosting Classifier (RFB)

As mentioned in the previous section, the Random Forest with Boosting (RFB) is used as a classifier [39,40]. The RF helps in making the different tress with different thresholds of instances. The RF is not able to make decisions effectively with the combination of various trees; therefore, the effectiveness ratio of trees is calculated as follows:

m=1,2,……..M

(17) $$\;tree_{m}\leftarrow Learn\left({\left\{x_{i}-\frac{\partial L\left(y_{i},F\left(x_{i}\right)\right)}{\partial F\left(x_{i}\right)}\right\}}_{i=1}^{n}\right)$$

(18) $$F\left(x\right)\leftarrow F\left(x\right)+\eta tree_{m}\left(x\right)$$

In the equation, ${\left(x_{i},y_{i}\right)}_{i=1}^{n}$ represents the training set, L(y,F(x)) is the loss function, η represents the learning rate, which is equal to 0.3, M is the number of tree (iterations) and F(x) is the model that is initialized by zero.

The key objective of the RF is to make all possible tress from the features with their respective severity classes, and the boosting approach provides the threshold value of the model for selection of the tree at the testing phase. This threshold value varies in the execution of the training phase, expressed as follows:

(19) $$F\;(x)\;=\;\mathrm{sign}\;(\sum_{t=1}^{T}\alpha_{t}h_{t}\left(x\right))$$

In Equation (19), *F* (*x*) represents the final threshold of the model and x shows the tree value; *αt* and *ht* represent the hyper parameter, which externally changes the threshold, and this output goes to sign (sigmoid function). The output of the RFB is considered as the final severity class of the bug report.

## 4. Experimental Setup

The platform of the experiment is a personal computer equipped with an Intel Core i5 (7th generation), with 8 GB of DDR4 memory and an NVIDIA GEFORCE GPU, and the operating system is Windows 10 with 64-bit, 2.70 GHz CPU. The CNN is implemented using Keras and TensorFlow, a Python library that bolsters easy implementation of CNN with the capacity of using both CPU and GPU to accelerate the network training. 

### 4.1. Dataset

In this work, the five open source datasets are used [26] from different projects, which have a different number of severity classes, as shown in Table 1.

### 4.2. Parameter Settings

The BCR model should be compiled for adequate training, which outlines the procedure of discovering the best weight sets to perform severity classification. The compilation of the BCR model requires the stipulation of the lost function that is employed to evaluate the weight set, the optimizer that is utilized to examine the different weights towards the network, and the metrics that is adapted to analyze the model performance. The loss function is similar to the metrics, but the outcomes that are evaluated by the metrics are not utilized for BCR model training, while the loss function is reduced by the optimizer to enhance the BCR model during the training. In this research paper, the cross-entropy has been used for loss function, and gradient decent for the optimizer and accuracy of metrics. After the efficient computation, the BCR model is compiled on training samples for the training procedure. The training procedure runs for 10 epochs and 150 iterations, which is stipulated by the epoch parameter. Further, the 150-batch size is used to evaluate the number of samples before updating the weights in the network. The errors and the trials decide the batch size and epoch. The feature extraction model and classifier training are generally offline processes and do not contribute to the testing time. Take as an example the Firefox dataset; the training time of the BCR is about 250 s per epoch. The entire testing and training time of the classifier is 300 s. After the training, a new bug report takes 14 milliseconds for classification (feature extraction + classification).

## 5. Results

The performance of the proposed BCR model was evaluated by the experiment on five different datasets, which are Mozilla, Eclipse, JBoss, OpenFOAM, and Firefox, which have a different number of severity classes, as shown in Table 1. The proposed approach is compared with the existing machine learning algorithm [26]. The data set was divided by using a 10-fold cross-validation. The widely used performance metrics were adopted to evaluate the classification accuracy: F-measure, Precision, and Recall [41].

RQ1. Is it feasible to implement an automated multiclass bug severity classification model using deep learning techniques, and what is the best configuration of Bug Severity Classification via the Convolutional Neural Network and Random Forest (BCR).

**Figure 5:**
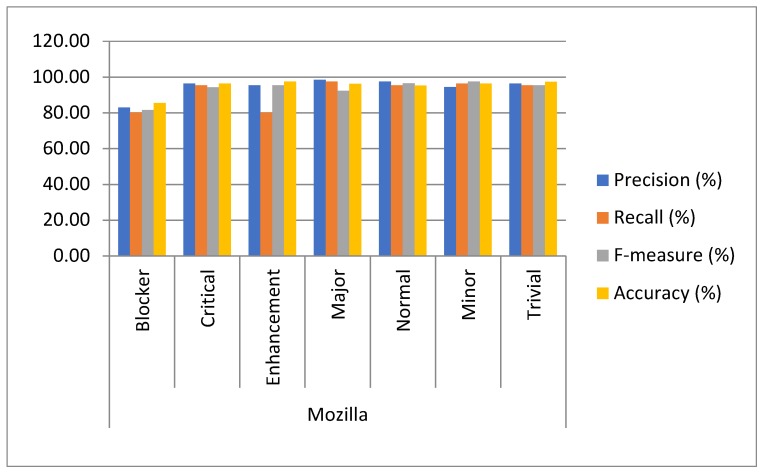
Results analysis of the proposed BCR on seven classes of the Mozilla Project.

**Figure 6:**
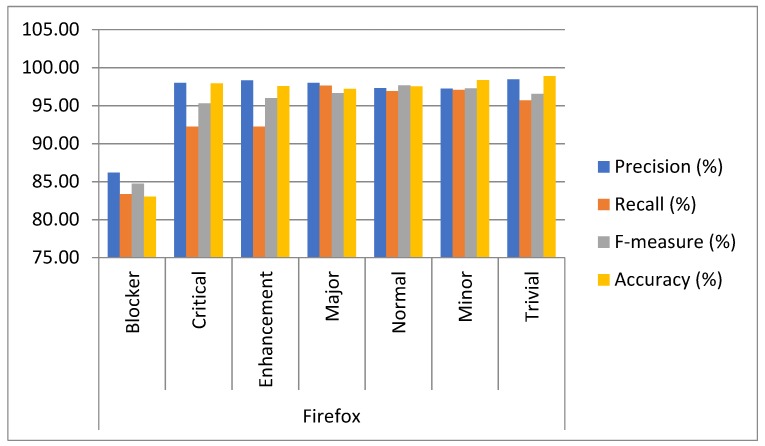
Results analysis of the proposed BCR on seven classes of the Firefox Project.

**Figure 7:**
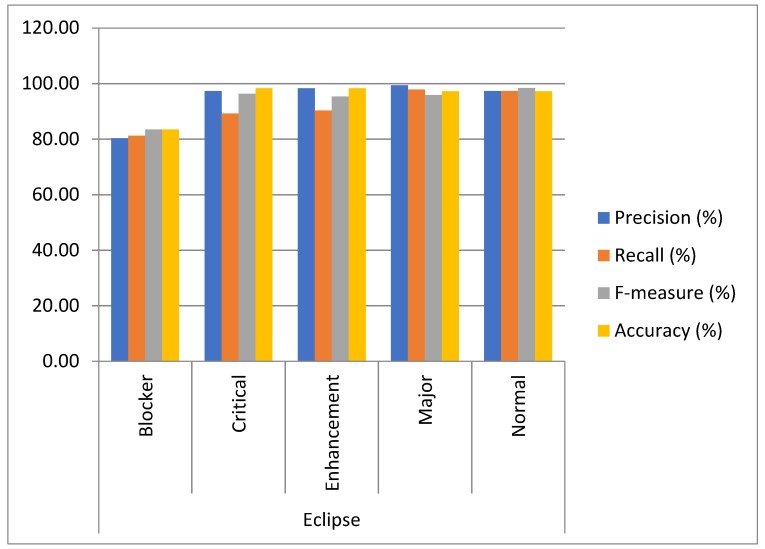
Results analysis of the proposed BCR on seven classes of the Eclipse Project.

**Figure 8:**
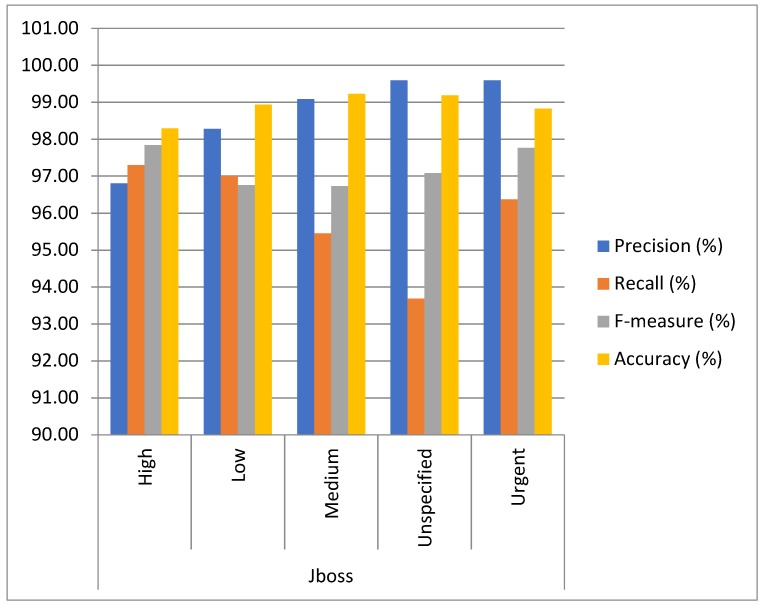
Results analysis of the proposed BCR on seven classes of the Jboss Project.

**Figure 9:**
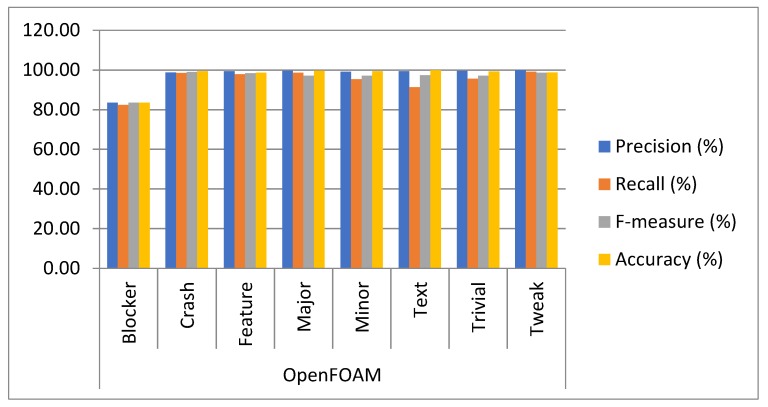
Result analysis of the proposed BCR on seven classes of the OpenFOAM Project.

**Figure 10:**
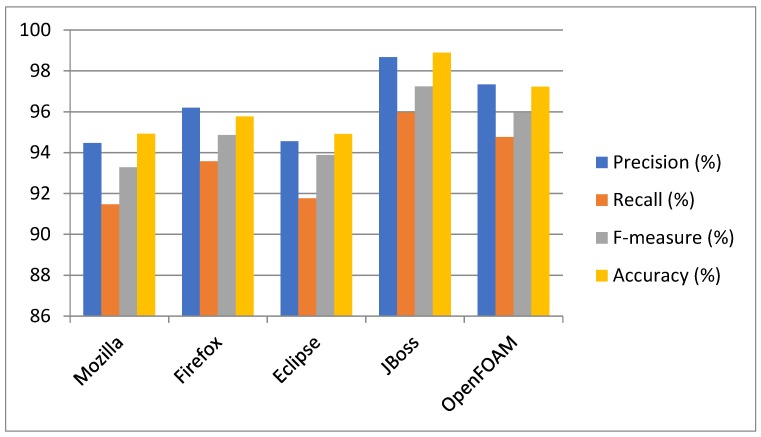
Comparison of the proposed BCR on all classes and datasets.

Approach: The experiment was conducted on five datasets by using 10-fold cross-validation to determine the capacity of multiclass bug severity classification with the same parameter settings. The method of experimentation is illustrated in Section 3. The average as well as individual accuracy and F-measures of each severity class of each dataset are calculated to evaluate the BCR model performance, as shown in Table 2 and Table 3 and Figure 5, Figure 6, Figure 7, Figure 8, Figure 9 and Figure 10.

Findings: It can be seen from the obtained results (Table 2 and Table 3) that it is feasible and probably competent to implement the deep learning technique for multiclass bug severity classification. The proposed BCR model provided the average accuracies and F-measures for Mozilla, Eclipse, JBoss, OpenFOAM, and Firefox in the range of 94% to 97% and 93% to 96%, respectively. The Mozilla dataset has seven severity classes, which are Blocker, Critical, Enhancement, Major, Normal, Minor, and Trivial. Table 2 depicts the accuracies and F-measures of seven classes, which are in the range of 85% to 98% and 81% to 96%, respectively. As we can see the Blocker class has low accuracy and F-measure, which are 85.45% and 81.45%, respectively, compared with the other severity classes. The critical class has 96.33% accuracy and 94.34% F-measure. Similarly, the Enhancement, Major, Normal, Minor, and Trivial classes have accuracies of 97.43%, 96.30%,95.24%,96.34%, and 97.33%, respectively, and F-measures of 95.45%, 92.34%, 96.45%, 97.44%, and 95.33%, respectively. This means that the performance of the proposed BCR model is not sufficient for the Blocker severity class. In contrast, the proposed model is sufficient for other severity classes for the Mozilla dataset. It can be inferred from Table 2 that the performance of the model for the Firefox dataset is somewhat consistent in classifying the severity of the Blocker class and other classes. This is evident in the accuracies and F-measures of seven classes in the ranges of 83% to 99% and 84% to 97%, respectively. The accuracies of the Blocker class are 83% and 97.92%, 97.55%, 97.19%,97.53%, 98.33%, and 98.35% for critical Enhancement, Major, Normal, Minor, and Trivial classes, respectively. Thus, it can be concluded that the BCR model performed very well in terms of critical Enhancement, Major, Normal, Minor, and Trivial classes, but not for the Blocker class. Table 2 also shows the results of the Eclipse dataset, which has five severity classes (Blocker, Critical, Enhancement, Major, and Normal) and has accuracy and F-measure in the ranges of 83% to 98% and 83% to 99%, respectively. The blocker severity class has low accuracy as compared to other severity classes. The proposed BCR model performed consistently well in classifying the severity of bug reports for other classes but did not work well for the Blocker class. The OpenFOAM dataset has eight classes (Blocker, Crash, Feature, Major, Minor, Text, Trivial, and Tweak) and its accuracy and F-measure are in the range of 83% to 99%. However, for the Blocker severity class, the accuracy is 83.40%, which is lower than other severity classes. Thus, consistent performance of the BCR model is indicated. This is because the given dataset has a limited number of bug reports for the Blocker class. Therefore, there is less data available for training processes and features do not fit well enough to get better classification. For example, the Mozilla dataset has 539 bug reports. The bug reports are classified into the seven classes. Out of the 539, the 218 bugs come under the Blocker, Critical, and Major categories. These bug reports have some serious issues, such as crashing, loading, or triggering of the application, and the e-commerce application does not correctly tally totals and subtotals. Out of 218 bugs, the Blocker class contains only 40 bug reports, Critical class contains 89 bug reports, and Major class contains 85 bug reports. The remaining 321 bug reports come under Minor, Enhancement, Normal, and Trivial classes. These bug reports have some cosmetic or design features, such as the application not meeting certain criteria or still exhibiting some unnatural behavior, an image being out of alignment, and so on. The bug reports that come under the Blocker class are fewer in number. Further, The JBoss dataset has five classes, which are High, Low, Medium, Unspecified, and Urgent, and the accuracy and F-measure are in the ranges of 98% to 99.22% and 96% to 98%, respectively. It can be concluded that the performance of the BCR depends on the data quality, which can vary in open source datasets. Further, some classes obtained low classification accuracy as compared to other classes in five open source projects. This is because the classification accuracy depends on the volume of the training data for every class. In this paper, the minimum number of classes is five and the maximum is eight. Therefore, the bug report dataset has less data to specifyeach class for the classifier to differentiate among them. Further, most of the bug reports are not detected with the severe issues that could harm the entire functionality of the system or the application. Infact, they belong to the minor changes, such as spelling mistakes, a link not working properly, or cosmetic or design errors. The recent studies have already discussed that the large amount of bug reports are in reality non-relevant in relation to the severe bugs [26]. The dataset used in this work has a notable amount of non-severe bugs. Thus, the proposed BCR model has allowed for a composition of severity classification models of each classification model tuned for the characteristics of the underlying data. The results show the feasibility and effectiveness of the BCR model. The F-measure and accuracy of the BCR model are not incremental in relation to the number of the convolutional layers. In our experiment, seven layers are sufficient to crest the BCR model with hybrid activation functions. The increment in the pooling layers promotes the mislaying of long term-dependencies (LTD) and local information. Therefore, the best configuration of the BCR is described in Table 4.

RQ2. Does the proposed algorithm outperform the traditional machine learning algorithms in bug severity classification?

Approach: The proposed deep learning model is compared with the existing machine learning model [26] based on bug severity classification. Zhou et al. proposed a hybrid approach by combining text and data mining models. They applied the Multinomial Naive Bayes (MNB) algorithm for binary severity classification. They classified the bugs into either bug or non-bug. Further, the authors validated the proposed approach on five open source projects, as shown in Table 1. To compare the efficiency of the proposed BCR model, the average F-measure of the binary classes (bug or non-bug) was computed on the same dataset as used by Zhou et al. 

**Figure 11:**
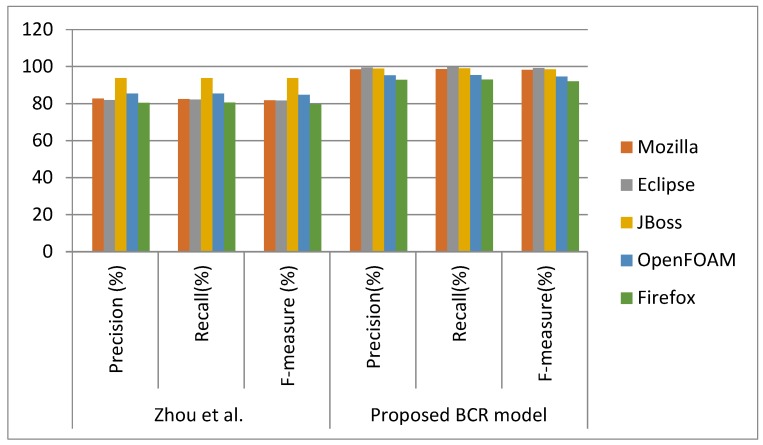
Comparison of the proposed and existing bug severity classifier on binary classes.

Finding: The comparison is shown in Table 5 and Figure 11. Zhou et al. have shown the average F-measure of binary classes is in the range of 79% to 94% for all five projects. It can be seen that the average F-measure of the BCR of the binary class is between 91% to 99%, and this highlights the ability of the extensive features extracted by the FECNN model. It is also clearly apparent that BCR significantly outperforms the existing machine-learning-based bug severity classification model. The total average F-measure of BCR reaches 96.43% for five projects, which is roughly 13% higher than the average F-measure of the existing machine learning bug severity classification model. This is because the CNN model automatically detected the highly complicated and important non-linear features in some initial hidden layers, whereas the shallow machine learning algorithms used the hand-crafted filter for feature extraction for learning. Further, random forest with boosting classified the more complex data rather than shallow techniques by utilizing the features that are extracted by the FECNN model.

## 6. Threats to Validity 

In this research work, there are specific threats to authenticate the validity of the BCR model results. The learning-based severity classification model has some common threats, while some of them are peculiar to our research problem, which are given below:

- The results are manifested using five bug repositories with distinct characteristics to assure generalization. Further, commercial BTS may accompany different patterns, and hence the BCR model outcomes may not be legitimately extensible to such bug repositories;
- Currently, in the BCR model, only the descriptions and titles of bug reports are examined. While these two-unstructured data are significant, as shown by the experiments, there exists other structured data that are required to classify the bug severity sufficiently;
- Generally, most of the bug reports are misclassified. Therefore, the experiment is conducted on manually labeled bug datasets from the actual paper. Although the actual paper proposes some rule sets to support their severity classification, errors might occur. These rules and labeling process depend on a personal perspective;
- Assuring that the statistical methods are reliable is yet another challenge. From this, the collinear variables are removed to confirm that the bug report data is not inflated. The statistical tests are applied, and the bug report dataset is adjusted by using stratified sampling;
- The other step is to assure that the BCR model does not experience overfitting, therefore the K-fold-cross-validation is applied, where each sample is used for both training and testing, thus providing a more accurate model.

## 7. Conclusions

Bug severity is a crucial attribute of the reported bug report, which is required to be resolved quickly after the reporting. All of the bugs do not relate to critical issues; some are frivolous and only require improvement. However, manual severity classification is often tedious and time-consuming. The convolutional neural network is successful in text classification; a new deep learning model has been proposed, called Bug Severity Classification via Convolutional Neural Network and Random Forest with Boosting (BCR).As per this model, firstly, the text of a bug report is preprocessed, followed by the application of the n-gram technique to extract the n-gram, then the CNN is applied to extract the latent and high representative features, and eventually, the random forest with boosting is applied to classify the multiple bug severity classes. The BCR model has been validated on five real datasets. The performance of the BCR is compared with the state-of-the-art technique for binary bug severity classification. The results show that the BCR model classifies the bug severity more accurately and effectively. The BCR model significantly improves the average f-measure in comparison to the recent work on bug severity classification. In the future, we will validate other deep learning approaches on more open source projects.

## Figures and Tables

**Table 1:** Bug report dataset.

Projects	Number of Bugs	BTS	Number of Classes	Name of Severity Classes
Mozilla	539	Bugzilla	7	Blocker, Critical, Enhancement, Major, Normal, Minor, Trivial
Eclipse	693	Bugzilla	5	Blocker, Critical, Enhancement, Major, Normal
JBoss	573	RedhatBugzilla	5	High, Low, Medium, Unspecified, Urgent
OpenFOAM	795	Manits	8	Blocker, Crash, Feature, Major, Minor, Text, Trivial, Tweak
Firefox	620	Bugzilla	7	Blocker, Critical, Enhancement, Major, Normal, Minor, Trivial

**Table 2:** Analysis of the proposed BCR model on five projects.

Projects	Classes	Precision (%)	Recall (%)	F-Measure (%)	Accuracy (%)
Mozilla	Blocker	82.90	80.23	81.54	85.45
	Critical	96.43	95.34	94.34	96.33
	Enhancement	95.33	80.22	95.45	97.43
	Major	98.45	97.45	92.34	96.30
	Normal	97.44	95.34	96.45	95.24
	Minor	94.35	96.34	97.44	96.34
	Trivial	96.35	95.34	95.33	97.33
Firefox	Blocker	86.16	83.35	84.73	83.00
	Critical	97.97	92.23	95.27	97.92
	Enhancement	98.30	92.23	95.98	97.55
	Major	97.98	97.61	96.64	97.19
	Normal	97.28	96.90	97.64	97.53
	Minor	97.23	97.04	97.24	98.33
	Trivial	98.43	95.68	96.52	98.85
Eclipse	Blocker	80.34	81.23	83.45	83.45
	Critical	97.33	89.23	96.35	98.33
	Enhancement	98.34	90.29	95.35	98.32
	Major	99.40	97.85	95.85	97.22
	Normal	97.35	97.29	98.40	97.24
Jboss	High	96.80	97.29	97.84	98.29
	Low	98.27	97.00	96.75	98.93
	Medium	99.08	95.45	96.72	99.22
	Unspecified	99.59	93.68	97.08	99.19
	Urgent	99.59	96.37	97.76	98.82
OpenFOAM	Blocker	83.45	82.34	83.45	83.40
	Crash	98.70	98.42	98.89	99.38
	Feature	99.28	97.81	98.33	98.56
	Major	99.61	98.51	97.11	99.54
	Minor	99.06	95.29	97.02	99.28
	Text	99.29	91.21	97.30	99.77
	Trivial	99.45	95.52	97.05	99.22
	Tweak	99.82	99.02	98.57	98.68

**Table 3:** The average result of all datasets.

Approach	Result Analysis				
	Projects	Precision (%)	Recall (%)	F-Measure (%)	Accuracy (%)
Proposed BCR	Mozilla	94.47	91.46	93.27	94.92
	Eclipse	96.19	93.57	94.86	95.76
	JBoss	94.55	91.76	93.88	94.91
	OpenFOAM	98.67	95.96	97.23	98.89
	Firefox	97.33	94.76	95.96	97.22

**Table 4:** The configuration of the BCR model.

Layer	Operator	Output Height	Output Width
Input	1000 × 200	1000	200
Dropout	Rate = 0.2		
Convolutional	Stride = 1, padding = 0, depth = 128, filter size = 20; activation = sigmoid for first four layers, Tanh for last three layers	128 64 32	128 64 32
Pooling	Max pooling		
Fully connected layer	Output depth = Random forest		

**Table 5:** Comparison of results of five projects based on binary classification.

Projects	Zhou et al.			Proposed BCR Model		
	Precision (%)	Recall (%)	F-Measure (%)	Precision (%)	Recall (%)	F-Measure (%)
Mozilla	82.60	82.40	81.70	98.48	98.52	98.12
Eclipse	81.80	82.10	81.60	99.38	99.48	99.12
JBoss	93.70	93.70	93.70	98.88	98.95	98.42
OpenFOAM	85.30	85.30	84.70	95.25	95.35	94.55
Firefox	80.30	80.50	79.50	92.75	92.95	91.95
